# Changing Landscape of Cancer Vaccines—Novel Proteomics Platform for New Antigen Compositions

**DOI:** 10.3390/ijms23084401

**Published:** 2022-04-15

**Authors:** Petr G. Lokhov, Steven Lichtenberg, Elena E. Balashova

**Affiliations:** 1 Biobohemia, Inc., 1 Broadway, 14th Floor, Cambridge, MA 02142, USA; sl@biobohemia.com (S.L.); balashlen@mail.ru (E.E.B.); 2 Institute of Biomedical Chemistry, 10 Building 8, Pogodinskaya Street, 119121 Moscow, Russia

**Keywords:** antigenic essence, upgrade, cancer vaccine, cell proteomic footprint, mass spectrometry, consortium

## Abstract

The creation of cancer vaccines is a constant priority for research and biotechnology. Therefore, the emergence of any new technology in this field is a significant event, especially because previous technologies have not yielded results. Recently, the development of a cancer vaccine has been complemented by a new proteomics technology platform that allows the creation of antigen compositions known as antigenic essences. Antigenic essence comprises a target fraction of cellular antigens, the composition of which is precisely controlled by peptide mass spectrometry and compared to the proteomic footprint of the target cells to ensure similarity. This proteomics platform offers potential for a massive upgrade of conventional cellular cancer vaccines. Antigenic essences have the same mechanism of action, but without the disadvantages, and with notable advantages such as precise targeting of the immune response, safety, controlled composition, improved immunogenicity, addressed MHC restriction, and extended range of vaccination doses. The present paper calls attention to this novel platform, stimulates discussion of the role of antigenic essence in vaccine development, and consolidates academic science with biotech capabilities. A brief description of the platform, list of cellular cancer vaccines suitable for the upgrade, main recommendations, limitations, and legal and ethical aspects of vaccine upgrade are reported here.

## 1. Introduction

Vaccines provide, after one or just a few injections, long-term protection to the body, making them exceptional among other drugs and widely used. The mechanism of action of conventional vaccines is well-understood. It is not surprising therefore that such a powerful tool has been aimed at fighting cancer. The first cancer vaccines were based on inactivated cancer cells, implementing the well-known and well-understood principle that you should vaccinate with what you want to develop protection against. Such vaccines have been developed against a significant number of cancers, including prostate cancer [1], lung cancer [2,3,4], colorectal cancer [5,6,7,8], melanoma [9,10,11], and renal cell cancer [12,13,14]. The clinical potential of such vaccines has been described, but none have yet passed the clinical trial stage [15,16].

Despite the persistent failure in trials of vaccines based on cancer cells, the development of such vaccines continues to occupy a leading position along with tumor-associated antigen (TAA)-based vaccines [17]. Vaccine trials still largely fuel the approach rather than discourage it, leading to new attempts with only marginal gains. Currently, several points supporting the development of new cellular cancer vaccines can be stated:

- A clear immunological basis, mechanism of action, and full-fledged source of the entire variety of native antigens are persistent drivers behind the creation of cellular cancer vaccines.
- Despite the development of many cell-based cancer vaccines, they do not pass clinical trials. The high activity of this direction is explained by the fact that cell-based vaccines are promising in their clinical trials but fail to meet the stringent requirements of regulators in terms of efficiency.
- Upgrading the existing cellular vaccines to address their shortcomings may allow developers to overcome common stumbling points and revitalize the field of cancer vaccines.

**Figure 1:**
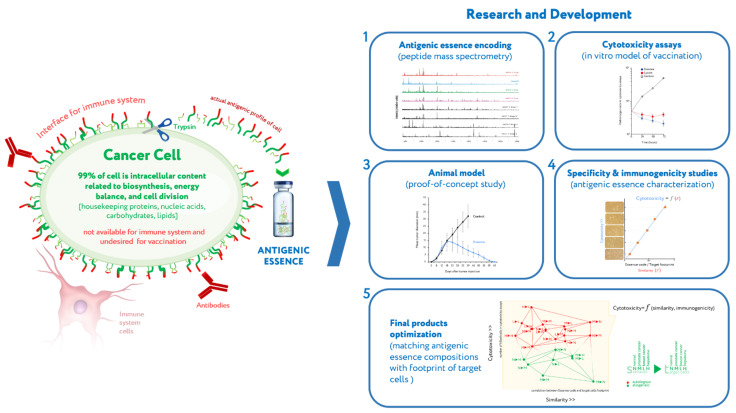
Summary of antigenic essence and its research and development (R&D). Actual antigenic properties of live cells are defined by the pool of antigens presented on the cell surface. Intracellular content is considered noise to be excluded from the vaccine composition. To this end, cells are treated with a purified protease (proteomics-grade trypsin) under mild conditions, and the released fragments of cell surface proteins are collected. The resulting antigenic essence is then (1) analyzed by mass spectrometry, tested both (2) in vitro and (3) in vivo, (4) characterized for specificity (as compared with the cell footprint of targeted cancer cells) and immunogenicity, and (5) optimized for final composition. The data presented in plots 1–5 are from the completed R&D studies. Adapted with permission from Ref. [18].

Given these points, it becomes clear that improvement of vaccines could radically change the situation. A new proteomics platform generating antigenic essence offers such an opportunity (Figure 1).

Briefly, cells are a natural source of a variety of antigens, but the use of whole cells has drawbacks. Only antigens on the cell surface are targets for vaccination, while antigens inside the cell can and should be ignored. Antigens on the cell surface are the only ones available to the immune system because the plasma membrane of living cells is impermeable to the cytotoxic elements of the immune system that recognize antigens, be they antibodies or cytotoxic lymphocytes. It should be noted that all extracellular antigens are produced inside cells and therefore can always be found there as well, and some intracellular antigens can be presented on the cell surface by major histocompatibility complex (MHC); antigens can be found anywhere within a cell, but they only act as immune targets when they are present on the cell surface. Therefore, the target pool of antigens located on the cell surface forms an actual antigenic profile of living cells. Given that the cell membrane is impermeable to macromolecules, this target pool of antigens can be collected by treating living cells with protease at mild conditions, thus yielding antigenic essence: the part of a cell that is both available to the immune system and highly specific to cell type on a molecular profile level. Research and development (R&D) of antigenic essence technology lasted fifteen years and covered antigenic essence production, composition analysis, investigation of immunogenic properties, proof-of-concept animal study, and design of final products which demonstrate optimized immunogenicity and similarity to target cells in tumors [19,20,21,22,23,24,25,26,27,28,29]. A description of the R&D conducted to date is beyond the scope of this article and can be found in a previously published review publication [18].

**Figure 2:**
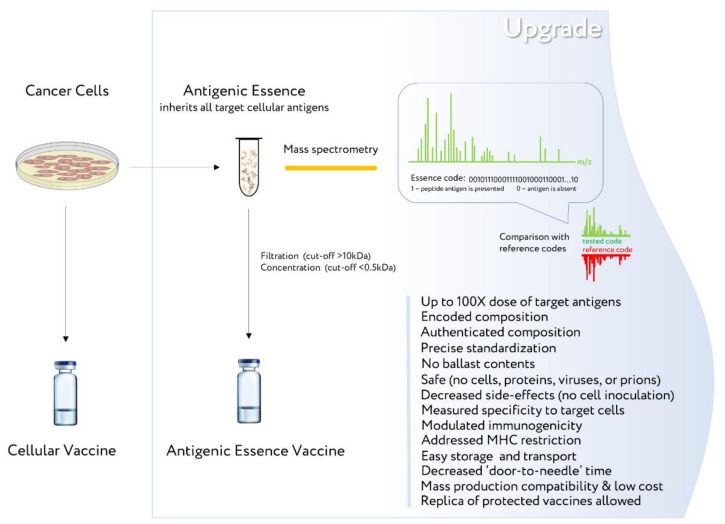
Upgrade of cellular cancer vaccines by preparing their antigenic essence analogs. The scientific background for such upgrade is provided in Ref. [18].

Antigenic essence inherits all the beneficial properties of cells—the full complement of native antigens—but is free of significant disadvantages. For example, it lacks cellular ballast (non-target antigens), offers precise control of antigen composition, addresses MHC restriction, and more. Therefore, it makes sense to upgrade the existing cellular cancer vaccines by replacing whole cells with the antigenic essence of these cells (Figure 2).

**Figure 3:**
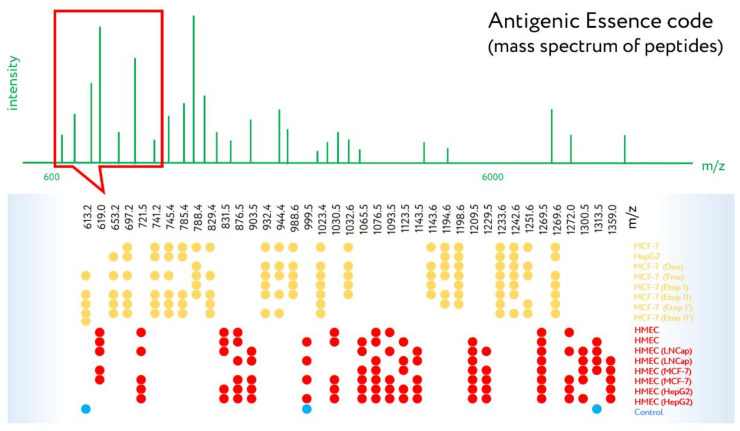
Mass spectrometry of antigenic essences. Antigenic essences are composed of tryptic peptides which are analyzed by peptide mass spectrometry. Shown are fragments of MALDI-TOF mass spectra which demonstrate the high specificity of antigenic essence compositions, both in terms of cell typing and relevance for vaccination subtypes. (●) m/z values of the quality control spectrum, which make it possible to detect the presence of impurities (e.g., trypsin autolysis products, contaminants). (●) m/z values for the antigenic essences of cancer cells (MCF-7 and HepG2) and drug-selected cancer cells (‘Dox’, ‘Tmx’, ‘Etop’—cells selected by doxorubicin, tamoxifen, and etoposide, respectively; I & II relates to single and double-selected cells with IC50 and IC95%). (●) m/z values for the antigenic essences of human microvascular endothelial cells (HMECs) obtained from two donors and stimulated by growth supplement or by stimuli from MCF-7, LNCap, or HepG2 cancer cells. Adapted with permission from Refs. [25,29].

The possibility of increasing the dose of target antigens, together with the other competitive characteristics of antigenic essence, will allow upgraded vaccines to achieve the necessary increase in efficiency to meet the requirements of regulators. The ability to specifically code their composition (Figure 3) makes it possible to systematize, standardize, and catalog them to make databases of vaccine codes, thus facilitating the coordinated mass creation of vaccines. The great similarity in the manufacturing of antigenic essence vaccines, as well as their ease of transportation and storage, will accelerate the development and distribution of cancer vaccines in an upgraded form. 

## 2. List of Vaccines Suitable for the Upgrade

Antigenic essence can be prepared for any cells used in a cellular vaccine, but an upgrade of tested in clinical trials cellular vaccines are prioritized. A shortlist of such cellular vaccines suitable to be upgraded is offered in Table 1. Since the history of vaccine development spans several decades, the table content is far from the complete list; rather, it focuses on relatively recent allogeneic cell-based vaccines. Although autologous vaccines are also eligible for an upgrade, allogeneic ones would be able to be upgraded more quickly and are therefore first in line. The same rule should be applied for upgrading cell vaccines that are not presented in the table. In the case of vaccines made with autologous dendritic cells, loading the dendritic cells with allogeneic antigens will also be a priority. 

Attention should be paid to vaccines intended for upgrade which consist of genetically modified cancer cells with increased expression of oncoantigens. Increased expression of surface antigens fits well into the concept of an upgrade since the antigenic essence accordingly will be enriched with such antigens. A notable example is BriaVax (BriaCell Therapeutics Corp., West Vancouver, Canada), prepared from a breast cancer cell line that expresses the surface protein Her2/neu, which is overexpressed in breast and ovarian cancers [74].

It is important to consider the cause of failure of cellular vaccines in clinical trials. Typically, the primary endpoint of improving overall survival (OS) or similar was not met, and in this context vaccine dosage is likely the issue. For example, the median OS of GVAX cancer vaccine was 24 months (low-dose) and 35 months (high-dose) in one study [63], and 23 months (low-dose) and 35 months (high-dose; equal to 3× low-dose) in another study [65]. This indicates the dose-dependent nature of the effectiveness of cell-based vaccines. In terms of the number of cells used for vaccination, antigenic essence allows for 100× the dose, without exceeding antigen limits or introducing side effects typically associated with cell-based inoculation. If indeed the failure of cellular vaccines is based on an insufficient increase in OS, then it is highly likely that upgraded vaccines will succeed in clinical trials. It is recommended to initially focus on this parameter as the main one, and then follow up with the remaining advantages of the technology (vaccine safety, standardization, composition control, etc.,).

It is important to note that, since antigenic essence comprises about 1% of the total cell protein content, its use allows up to 100 times more cells and, therefore, target antigens to be used for vaccination as compared to whole cells. However, vaccination with very low amounts of antigenic essence may be also important because high doses of antigens have been shown to yield lower-quality antibodies, especially in terms of affinity [75,76,77]. High avidity T cells are capable of being stimulated by extremely low concentrations of antigen [78,79,80,81], and it has been shown that T cells with higher functional avidity are more effective at treating tumors [81,82,83].

**Figure 4:**
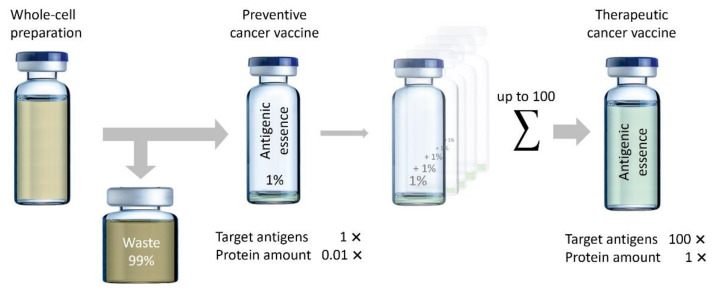
The creation of preventive and therapeutic vaccines based on antigenic essence. Preventive vaccines have the same amount of target antigens (1×) but only 0.01× of the total protein level contained in the original whole-cell vaccines, resulting in a more functional immune response and lower side effects. Therapeutic vaccines, being composed only of antigenic essence, do not exceed the total protein level of the original whole-cell vaccines (1×) but can be up to 100× of the dose of target antigens.

In addition to the fact that higher doses of antigen may lead to lower avidity of T cells and antibodies, the use of high-dose antigens can also lead to (i) more adverse events, (ii) tolerance of the targeted T cells [77], (iii) clonal deletion via apoptosis, especially of high avidity T cells [79,80], and (iv) exhaustion of T cells [84]. Therefore, the prevailing concept of “the more antigens, the better” is not always justified. It seems reasonable to develop cancer vaccines based on small doses of targeted antigens that will not have a significant negative impact on the immune system (Figure 4). Thus, antigenic essence may influence the development of preventive cancer vaccines, which are used in disease-free people and thus have stricter safety requirements.

It should be mentioned that whole-cell vaccines for which only R&D data are available may also be upgraded. Such R&D data, which is often costly to obtain, can be used as supporting data that will facilitate the regulatory authority’s registration of updated vaccines as an innovative new drug (IND). Finally, antigenic essence technology makes it possible to create new vaccines that have no cellular analogs at all.

## 3. Limitations

The ability to encode antigenic essence by mass spectrometry and to assess its similarity to the antigenic profile of target cancer cells is one of its critical advantages [18]. However, upgrading cellular vaccines to antigenic essence-based vaccines uses the same cells. Therefore, the technology of antigenic essence is fully disclosed only when antigenic essence composition is precisely tuned to target cancer cells in the human body, and not when it is inherited from an existing whole-cell vaccine. Thus, the first antigenic essence vaccines will be limited by the composition of existing whole-cell vaccines. But the relative simplicity and low cost of obtaining them, together with the advantages of antigenic essence itself (Figure 2), more than justify their production. The next obvious stage in the development of cancer vaccines will involve matching the composition of antigenic essences with target cancer cells. Although this will complicate the process of obtaining vaccines, it will also reveal the vast technological potential of antigenic essence and open a new realm of possibility for effectiveness of cancer vaccines. An example of such a novel vaccine is known under the SANTAVAC name [28].

Despite the limitations, significant advantages allowed the developers of antigenic essence technology to declare it as a potentially trending technology demonstrating significant competitive features in comparison with the creation of vaccines based on whole cancer cells (Table 2).

**Figure 5:**
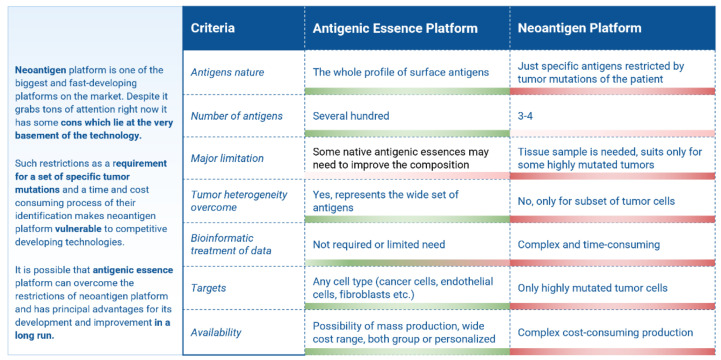
Cancer vaccines platforms comparison: Antigenic essence *versus* neoantigens. Adapted with permission from Ref. [18].

Antigen essence technology, being an improvement in the preparation of cancer vaccines, is interesting to compare with the current trend in cancer vaccines development based on neoantigens. Neoantigens arising from mutated proteins in cancer cells, are specific to each cancer, and their diversity allows researchers to select the most immunogenic among them, potentially bypassing MHC restrictions and allowing for use in immunotherapy. Major hurdles for neoantigen vaccination include the lengthy and cumbersome process necessary for the identification and selection of neoantigens, the exclusively personalized approach, and the limited number of tumors that possess enough mutations to apply this technology [87]. The comparison shows that the antigen essence platform can overcome the restrictions of the neoantigen platform and has principal advantages for its development and improvement (Figure 5).

## 4. Legal and Ethical Aspects of Cellular Vaccines Upgrade

This issue concerns many already developed cell vaccines, as well as those that are currently undergoing clinical trials or are still in the R&D stage. Such developments are lengthy and expensive. The average cost for the creation of one drug today is $1.3 billion [88]. Therefore, the question arises whether the appearance of improved analogs, possessing the same therapeutic properties as the cellular vaccines, violates the rights of the original developers. The developers of antigenic essence technology believe that this will not be an issue. Since antigenic essence technology has already been patented in many countries, patent authorities recognize its novelty, inventive step, and industrial applicability. Thus, from a legal point of view, antigenic essence preparations are not cellular vaccines, and their use is not limited by the presence of their cellular counterparts.

In addition to the legal basis of the application of antigenic essence technology, the ethical aspects should also be mentioned. The creation of analogs of cell-based vaccines is ethical in that it reflects progress. In many ways this is reminiscent of the emergence of CAR-T technology, which allowed TAAs to bypass MHC restriction, leading to the efficient re-use of well-known TAAs and a new trend in immunotherapy [89]. Second, if protected cell lines previously involved in cellular vaccine production are later used to produce antigenic essence, then the interest of the owners of such cell lines can also be considered through the purchase of licenses for their use. Finally, many start-up companies that have promoted cellular vaccines have long since closed after failing clinical trials. So, reviving their vaccines as an updated counterpart does not violate anyone’s rights.

## 5. Manufacturing

Contract development and manufacturing organizations (CDMO) and contract manufacturing organizations (CMO) serve other companies in the pharmaceutical industry on a contract basis to provide services from drug development to manufacturing. These organizations are quick to meet halfway, offer their services, and communicate execution time. However, these services usually involve known technologies that are implemented routinely; for example, the production of mRNA vaccines, cellular vaccines, or vaccines based on synthetic peptides. Requests for the production of antigenic essence usually confuse them. 

**Figure 6:**
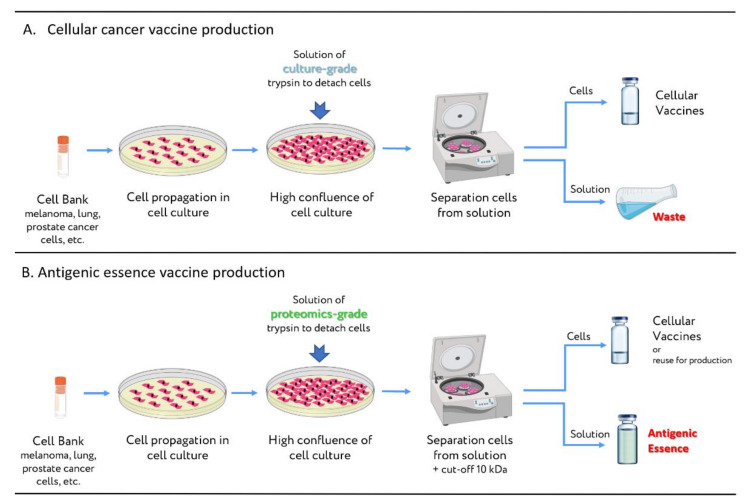
A simple modification allows the workflow for producing cellular vaccines to be used for producing antigenic essence. (**A**) Conventional workflow for manufacturing cellular vaccines. Cancer cells are propagated, and *culture-grade* trypsin is used to detach cells from the surface. The cellular preparation is obtained after discarding the trypsin solution. (**B**) Modified workflow for manufacturing antigenic essence. The key change is that *culture-grade* trypsin is replaced with *proteomics-grade* trypsin (more purified and protected from autolysis). Antigenic essence is obtained by filtering the used trypsin solution (cut-off 10 kDa) to remove trypsin. The remaining cellular preparation may be used to produce additional antigenic essence in future.

However, the process of antigenic essence production is well-known to CDMOs or CMOs dealing with cell cultures. It can be considered as a spin-off product of cellular vaccine production from adherent cell cultures (Figure 6). Briefly, cell cultures are grown to a certain density. Then cells are treated with culture-grade trypsin, the purpose of which is to sever the extracellular proteins responsible for cell surface attachment. The detached cells are separated from the trypsin solution by centrifugation, and are then used to formulate cellular vaccines. The used trypsin solution (which contains antigenic essence along with an admixture of crude culture-grade trypsin) is disposed of as waste. So, it can be claimed that CMOs and CDMOs constantly generate vast amounts of antigenic essence in solution, but all of it is disposed of. To obtain an antigenic essence product suitable for vaccination, manufacturing organizations need only replace the culture-grade trypsin with proteomics-grade trypsin, which is free of impurities and protected from self-destruction. The antigenic essence can then be purified from the used proteomics-grade trypsin by filtration (cut-off 10 kDa). Thus, replacing only one reagent with its higher-quality analog in the production of cellular vaccines turns waste into an innovative product.

In fact, from the perspective of antigenic essence technology, the production of cellular cancer vaccines is perverse: the waste is a valuable innovative product, while the product itself—the cellular preparation—is most likely waste. However, introducing antigenic essence production as a spin-off of cellular vaccine preparation will make it more accessible and less expensive. 

## 6. Consortium

Vaccines upgrade aligns with cancer research priorities in the USA, as declared by the Blue Ribbon Panel (e.g., “Development of new enabling cancer technologies”) [90]. The US National Cancer Institute is already implementing some of those recommendations. Following the same recommendations regarding the generation of science and trials networks, a consortium within the framework of the vaccines upgrade will be organized. The supporting pillars of that mission are to: 

a. Establish a network of collaborations to enable development of infrastructure, resources, and funding opportunities that will ensure sustained growth of the vaccines upgrade initiative. 

b. Promote education to enable scientists to work effectively with antigenic essence data, and for basic researchers to collaborate with oncologists. 

c. Provide a unified voice for the views and concerns of vaccines upgrade participants.

## 7. Conclusions

To a large extent, vaccines upgrade is based on the hopes and expectations of the developers of antigenic essence technology, to whom the advantages seem obvious. However, the participation of other scientists would accelerate and stabilize the process of antigenic essence adoption. Regarding the prematurity and speculativeness of vaccine upgrade, doubters can look at the status of those cellular cancer vaccines that may be upgraded (Table 1). None of these vaccines have passed clinical trials, yet they remain at the forefront of the vaccine industry. In this light, the development of novel, upgraded vaccines seems much less speculative than the continued development of a failed approach. Hence our motivation for alerting the scientific and biotech communities about the potential of this novel proteomics platform for generating cancer vaccines. 

## Figures and Tables

**Table 1:** Shortlist of cellular cancer vaccines suitable for the upgrade.

	Cellular Vaccine (Sponsor, Developer)	Description	Cancer Origin	Phase	Reference/Clinical Trial ID
1	CanVaxin (CancerVax Corp.; John Wayne Cancer Institute)	Administration of 3 allogeneic melanoma cell lines (M10-V, M24-V, and M101-V pooled in equal amounts) with BCG ^1^. Median OS ^2^: 12.9 months.	Colon	I	Habal, Gupta, et al. [30]
2	OncoVax (Vaccinogen Inc)	Vaccination with autologous colon cancer cells mixed with live BCG. Significant improvement in overall and disease-free survival in the IIIa study.	Colon	III	Vermorken, Claessenet, et al. [31]; NCT02448173
3	ONYCR1-3 (ONYvax)	Allogeneic adenocarcinoma cell-based vaccines mixed with BCG or alum adjuvant.	Colon	I/II	NCT00007826 (Arm II and III)
4	HyperAcute-Breast cancer (NewLink Genetics Corporation)	Genetically modified allogeneic tumor cells expressing the xenoantigen αGal.	Breast	I/II	NCT00090480
5	Allogeneic GM-CSF-secreting whole-cell breast cancer vaccine (Sidney Kimmel Comprehensive Cancer Center at Johns Hopkins)	The GM-CSF ^3^-producing 3SKBR3-7 and 2T47D-V cells combined into a single vaccine formulation.	Breast	I	Emens, Armstrong, et al. [32]
6	Allogeneic cellular cancer vaccine (Robert W. Franz Cancer Research Center, Earle A. Chiles Research Institute)	Vaccination with MDA-MB-231, an HLA-A2(+), HER2/neu(+) allogeneic breast cancer cell line genetically modified to express the costimulatory molecule CD80 (B7-1).	Breast	I	Dols, Smith, et al. [33].
7	Allogeneic cellular cancer vaccine (Sidney Kimmel Comprehensive Cancer Center at Johns Hopkins; NCI)	Allogeneic GM-CSF-secreting breast cancer cell lines (SKBR3 and T47D) alone or with CY and DOX.	Breast	I	Emens, Asquith, et al. [34]; NCT00093834.
8	Allo GM-CSF-secreting vaccine (Sidney Kimmel Comprehensive Cancer Center at Johns Hopkins)	Allogeneic GM-CSF-secreting breast cancer vaccine (two parts 2T47D-V and one part 3SKBR3-7) with trastuzumab and cyclophosphamide.	Breast	II	Chen, Gupta, et al. [35]; NCT00399529
9	KS2422-vacc (University Hospital Tuebingen; Paul Ehrlich Institute, Langen)	Allogeneic breast cancer cell line, KS24.22, genetically modified to express CD80 and Her-2/neu.	Breast	I	Gückel, Stumm, et al. [36]; NCT01127074
10	BriaVax (BriaCell Therapeutics Corporation)	BriaVax is derived from a human breast cancer cell line (SV-BR-1-GM) that expresses the protein Her2/neu, which is overexpressed in some epithelial cancers like breast and ovarian cancers. It was designed to produce and secrete GM-CSF.	Breast Ovary	I/II	NCT03066947
11	O-Vax (AVAX Technologies)	DNP-modified autologous ovarian tumor cell vaccine. Median OS: 22.7 months.	Ovary	I/ II	Berd, Sato, et al. [37]; NCT00660101
12	Autologous DC vaccine (Edward Hirschowitz; University of Kentucky; NCI)	Autologous dendritic cells loaded with allogeneic non-small cell lung cancer cells (NSCLC ^4^ cell lines that over-expresses Her2/neu, CEA, WT1, Mage2, and survivin).	Lung	II	Hirschowitz, Foody, et al. [38]; NCT0010311
13	MelCancerVac (Herlev Hospital; University of Copenhagen)	Autologous dendritic cells pulsed with allogeneic melanoma cell lysate (MelCancerVac) in combination with the Cox-2 inhibitor of celecoxib.	Lung	II	NCT00442754
14	HyperAcute-Lung Cancer (tergenpumatucel-L) (NewLink Genetics Corporation; NCI)	The vaccine consists of genetically modified allogeneic NSCLC tumor cells with the αGal moiety on the cell surfaces.	Lung	I/II	Pruitt, Kirk, et al. [39]; Lai, Kolber-Simonds, et al. [40]; NCT00073398
15	1650-G vaccine (University of Kentucky)	Allogeneic NSCLC cell line 1650 mixed with GM-CSF.	Lung	I/II	Hirschowitz, Mullins, et al. [41]; NCT00654030
16	Viagenpumatucel-L (HS-110) (Heat Biologics)	Allogeneic vaccine derived from irradiated human lung cancer cells genetically engineered to continually secrete gp96-Ig.	Lung	I/II	NCT02117024
17	GVAX lung cancer vaccine (Southwest Oncology Group; NCI)	K562 cells genetically modified to secrete GM-CSF combined with autologous lung tumor cells. Median OS: 5.4 months.	Lung	I/II	Nemunaitis, Jahan, et al. [42]; NCT00074295
18	Belagenpumatucel-L (Lucanix) (NovaRx Corporation)	Administration of Belagenpumatucel-L (a cocktail of 4 irradiated allogeneic NSCLC cell lines transfected with TGF-β2 antisense transgene). Median OS in II trial: 14.5 months.	Lung	II/III	Nemunaitis, Dillman et al. [43]; Nemunaitis, Nemunaitis, et al. [44]; Giaccone, Bazhenova, et al. [45] NCT01058785; NCT00676507
19	Allogeneic tumor cell-based vaccine (H. Lee Moffitt Cancer Center and Research Institute; NCI; NIH)	Allogeneic lung adenocarcinoma cells are combined with a bystander K562 cell line transfected with hCD40L and hGM-CSF.	Lung	II	NCT00601796
20	Allogeneic B7.1/HLA-A1 (University of Miami)	Administration of irradiated whole-cell (AD100) allogeneic vaccine transfected to express B7.1 along with either HLA-A1 or HLA-A2. Median OS: 18 months.	Lung	I/II	Raez, Cassileth, et al. [46]; NCT00534209
21	Allogeneic vaccine (University of Miami)	Allogeneic tumor cells secreting endoplasmic reticulum-chaperone gp96-Ig-peptide complexes. Median OS: 16.5 months.	Lung	I	Raez, Walker, et al. [47].
22	CanVaxin (CancerVax Corporation)	Allogeneic whole-cell vaccine consisting of three melanoma lines combined with BCG as an adjuvant. In phase II median OS and 5-year rate of survival were significantly higher in stage III melanoma.	Melanoma	III	NCT00052156; NCT00052130
23	Melacine (Corixa Corporation)	Administration of Mel-D and Mel-S cell lysates (Melacine) with DETOX.	Melanoma	I	Mitchell, Kanmitchell, et al. [48]
24	Melacine (Corixa Corporation)	Administration of Melacine with CY and IFN-α i.v. after 4 doses of Melacine. Median OS: 12.5 months.	Melanoma	II/III	Vaishampayan, Abrams, et al. [49]; NCT00002767
25	Melacine (Corixa Corporation)	Melacine administration. Investigation of the impact of class I antigen expression on relapse-free survival after adjuvant therapy with the vaccine (5 years relapse-free survival).	Melanoma	III	Sosman, Unger, et al. [50]
26	Autologous DC vaccine (Centro de Investigaciones Oncológicas FUCA et al.)	Ex vivo loading of autologous DCs with antigens from apoptotic/necrotic allogeneic melanoma cells and subsequent adoptive transfer. Apoptotic-necrotic (Apo-Nec) tumor cells were prepared as a batch of four cell lines (MEL-XY1; MEL-XY2; MEL-XY3 and MEL-XX4).	Melanoma	I	Von Euw, Barrio, et al. [51].
27	A2/4-1BBL melanoma vaccine (Hadassah Medical Organization)	Vaccination with irradiated M20/A2B cells.	Melanoma	II/III	NCT01898039; NCT01861938
28	VACCIMEL (Laboratorio Pablo Cassará S.R.L. et al.)	Administration of Cyp followed by VACCIMEL (a mixture of 3 allogeneic cell lines IIB-MEL-J, IIB-MEL-LES, and IIB-MEL-IAN).	Melanoma	II/III	Mordoh, Kairiyama, et al. [52]; NCT01729663
29	VACCIMEL (Laboratorio Pablo Cassará S.R.L. et al.)	Administration of VACCIMEL with rhGM-CSF.	Melanoma	I	Barrio, De Motta, et al. [53]
30	Dendritic cell vaccine (Baylor Institute for Immunology Research)	Administration of autologous monocyte-derived DCs loaded ex vivo with killed allogeneic Colo829 melanoma cells and activated with GM-CSF, IL-4, TNF-α, and CD40 ligand. Median OS: 22.5 months.	Melanoma	I	Palucka, Ueno, et al. [54]
31	BIBW2 component A and B (Boehringer Ingelheim)	Allogeneic tumor vaccine BIWB 2 containing melanoma cells transfected with the human IL-2 gene.	Melanoma	I	NCT02203864
32	M-Vax (DNP-VACC) (AVAX Tech.)	DNP-modified autologous tumor cells. 5-year OS rate was 46%.	Melanoma	I/ II	David Berd [55].
33	Autologous dendritic cell-allogeneic melanoma tumor cell lysate vaccine (Jonsson Comprehensive Cancer Center; NCI)	Matured dendritic cells pulsed ex vivo with 3 melanoma cell line lysates (IDD-3).	Melanoma	II	Ribas, Camacho, et al. [56]; NCT00107159
34	CSF470 Vaccine (Laboratorio Pablo Cassará S.R.L.)	Allogeneic 4 lethally irradiated cutaneous melanoma cell lines (MEL-XY1, MEL-XY2, MEL-XY3, and MEL-XX4).	Melanoma	II/III	Aris, Bravo, et al. [57]; Mordoh, Pampena, et al. [58]
35	GVAX (Sidney Kimmel Comprehensive Cancer Center at Johns Hopkins)	Allogeneic pancreatic tumor cells transfected with a GM-CSF gene administered in combination with Ipilimumab (an antibody that blocks negative signals to T cells).	Pancreas	I	Le, Lutz, et al. [59]; Hopkins, Yarchoan, et al. [60]
36	PANC 10.05 and PANC 6.03 vaccines (Sidney Kimmel Comprehensive Cancer Center at Johns Hopkins; Viragh Foundation)	A pancreatic vaccine secreting a GM-CSF and consists of equal numbers of pancreatic cancer cells (Panc 6.03) and (Panc 10.05) into a single vaccine.	Pancreas	II	NCT01088789
37	GVAX (Sidney Kimmel Comprehensive Cancer Center at Johns Hopkins; NCI)	Allogenic pancreatic tumor cell vaccine transfected with GM-CSF used with cyclophosphamide.	Pancreas	II	NCT00727441
38	GVAX	Irradiated GM-CSF transfected allogeneic whole-cell tumor lines. Two pancreas cancer cell lines (PANC 10.05 and PANC 6.03) were combined. The median disease-free survival is 17.3 months with a median OS of 24.8 months.	Pancreas	I/II	Lutz, Yeo, et al. [61]; NCT00084383
39	GVAX	The first administration of an allogeneic prostate cancer cell lines (PC3 and LNCap) modified to secrete GM-CSF.	Prostate	I/II	Simons, Carducci, et al. [62]
40	GVAX	Administration of allogeneic prostate cancer cell lines (PC3 and LNCap) modified to secrete GM-CSF. Median OS: 34.9 months (high dose); 24.0 months (low dose).	Prostate	I/II	Small, Sacks, et al. [63]
41	GVAX	Administration of GVAX plus ipilimumab (fully human IgG CTLA-4 blocking Ab). Median OS: 29.2 months.	Prostate	I	Van den Eertwegh, Versluis, et al. [64]
42	GVAX	Administration of 2 allogeneic prostate-carcinoma cell lines (PC3 and LNCap) modified to secrete GM-CSF. Median OS: 35.0 months (high-dose); 20.0 months (mid-dose); 23.1 months (low-dose).	Prostate	I/II	Higano, Corman, Smith, et al. [65]
43	ONY-P1 (ONYVAX-P) (GemVax & Kael Co., Ltd.; NCI)	The vaccine is derived from three irradiated allogeneic prostate cancer cell lines that represent different stages of prostate cancer.	Prostate	II	Doehn, Torsten, et al. [66] NCT00514072
44	Allogeneic vaccine (Institut für Experimentelle Onkologie und Therapieforschung)	Administration of LNCaP modified using retroviral vector to secrete IL-2 and IFN-γ. Median OS: 32 months.	Prostate	I/II	Brill, Kuebler, et al. [67] Brill, Kuebler Pohla, et al. [68]
45	Allogeneic whole-cell vaccine (OnyVax Ltd.)	Administration of whole-cell vaccine consisting of a mixture of 3 prostate cancer cell lines (Pr1-4) along with Mycobacterium vaccine (SRL172).	Prostate	I/II	Eaton, Perry, et al. [69]
46	DC-APCC (Mayo Clinic; NCI)	Allogenic whole prostate carcinoma cell (APCC) vaccine co-administered with ex vivo generated dendritic cells.	Prostate	II	NCT00814892
47	Neuroblastoma vaccine (Baylor College of Medicine; Center for Cell and Gene Therapy, Baylor College of Medicine)	Vaccination with unmodified SKNLP, with gene-modified SJNB-JF-IL2 and SJNB-JF-LTN neuroblastoma cells.	Brain		NCT01192555
48	Gliovac (ERC1671) (Daniela A. Bota; University of California; Epitopoietic Research Corporation)	Autologous and allogeneic tumor cell vaccines against glioblastoma based on irradiated DNFB-modified tumor cells.	Brain	II	Schijns, Pretto, et al. [70]; NCT01903330
49	Autologous DC vaccine (Mayo Clinic; NCI)	Allogeneic glioma tumor lysate-pulsed autologous dendritic cell vaccine.	Brain	I	NCT03360708
50	RCC26/IL-7/CD80 vaccine (Charite’-University Medicine et al.)	Administration of renal cancer cell line (RCC26) genetically modified to express IL-7 and CD80 (B7-1). Median OS: 40 months.	Kidney	I	Westermen, Flörcken, et al. [71]
51	MGN1601 (Mologen AG)	Genetically modified allogeneic tumor cells for the Expression of IL-7, GM-CSF, CD80, and CD154.	Kidney	I/II	NCT01265368
52	Allogeneic myeloma GM-CSF Vaccine (Sidney Kimmel Comprehensive Cancer Center at Johns Hopkins; CellGen Corporation)	Allogeneic GM-CSF secreting myeloma vaccine in combination with lenalidomide.	Blood	II	NCT01349569
53	K562/GM-CSF vaccine (Sidney Kimmel Comprehensive Cancer Center at Johns Hopkins; Cell Genesis Inc)	The vaccine produced from chronic myeloid leukemia (CML) cell line modified to secrete GM-CSF administered with imatinib mesylate.	Blood	I	Smith, Kasamon, et al. [72]
54	Allogeneic tumor cell vaccine (K562) (NCI)	Allogeneic tumor cell vaccine produced from cell line K562.	Lung Esophagus Pleura Thymus	I	NCT01143545
55	ADKV (N.N. Petrov National Medical Research Center of Oncology)	Autologous dendritic cell vaccine (ADKV) loaded with allogeneic tumor lysate expression of cancer-testis antigens (CTA).	Soft tissue sarcomas	I/II	NCT01883518
56	Allogenic tumor cell vaccine (K562) (NCI; NIH Clinical Center)	The vaccine produced from irradiated K562 erythroleukemia cells expressing GM-CSF (K562-GM cells).	Sarcoma Melanoma Epithelium Pleura	I	NCT01313429
57	Tumor cell vaccine (Hadassah Medical Organization; International Center for Cell Therapy & Cancer Immunotherapy)	Vaccination with allogeneic tumor cell lines that share MHC determinants with the patient aiming to overcome the possible restriction of antigen presentation.	Solid tumors	II	NCT00148993
58	Antiangiogenic cancer vaccine (University of Tokyo)	Vaccine using glutaraldehyde-fixed human umbilical vein endothelial cells (HUVECs).	Brain Colon	I	Okaji et al. [73]

^1^ BCG, Bacillus Calmette–Guerin; ^2^ OS, overall survival; ^3^ GM-CSF, granulocyte-macrophage colony-stimulating factor; ^4^ NSCLC, non-small cell lung cancer.

**Table 2:** Comparison of antigenic essence-based vaccines with whole-cell cancer vaccines.

Criteria	Antigenic Essence Vaccines	Whole Cancer Cell Vaccines (Irradiated Cells, Whole Cell Lysate or Fixed Cells)
Antigens	Full diversity of native antigens desirable for vaccination.	Full diversity of native antigens, a vast majority of which are intracellular antigens undesirable for vaccination.
Immunogenicity	Low (‘as is’), or decreased (compromised compositions ^1^), or increased [18,28].	Low (‘as is’), or increased (overexpression of some antigens and release of cytokines by irradiated cells); fixed cells have lower immunogenicity than whole cell lysate [85], irradiated cells have higher immunogenicity than whole cell lysate [86].
Bioinformatic processing of data	No, moderate, or enhanced processing of mass spectrometry data. ^2^	No
Target cell killing rate (in vitro)	Directly connected with antigenic essence composition [28,29].	There is no method to connect the antigen composition of whole cells with immune response.
Limitations	The lifetime of tryptic peptides is limited in the body; some epitopes may be fragmented, and the secondary structure of some peptide epitopes may be broken.	The vaccination dose includes all antigens of the cell, so less than 1% of the maximum allowed dose would include targeted antigens.
MHC-restriction	Not addressed or addressed ^2^ [18].	Not addressed.
Immunopeptidome	Enriched in comparison with whole cells [18].	As a trace amount in most other antigens.
Vaccine type	Preventive and therapeutic.	Only therapeutic.
Availability for mass production	Can be adapted for mass production (the same cancer cells can be used to produce antigens many times).	Cells for vaccination are used only once.
Stability	Stable (as peptide composition).	Unstable (as live or fixed cells, or as protein mixture).
Safety	High (no supramolecular structures, prions, or viruses present).	Low (tight control is required to exclude the presence of dangerous agents).
Quality Control	Improved (included control of antigen composition).	Moderate (no test related to control of antigen profiles of cells).
Clinical trials	Not yet conducted.	Failed in all clinical trials.

^1^ Antigenic essence composition compromises between specificity and immunogenicity. ^2^ Depends on the desired quality and complexity of the antigenic essence-based vaccine to be manufactured.

## Data Availability

Not applicable.
